# Risk factors and outcomes of delayed completion of the hexavalent 3 + 1 immunisation series in German preterm infants – an observational study

**DOI:** 10.1186/s40348-026-00228-1

**Published:** 2026-04-10

**Authors:** Hannah Kraft, Marie-Theres Dammann, Kathrin Hanke, Michael Zemlin, Janina Soler Wenglein, Jule Rohde, Désirée Lasserre, Alexander Humberg, Claudia Roll, Christoph Härtel, Jan Rupp, Folke Brinkmann, Sabine Pirr, Reinhard Jensen, Rainer Odendahl, Peter Ahrens, Christine Silwedel, Johannes Liese, Wolfgang Göpel, Egbert Herting, Guido Stichtenoth, Ingmar Fortmann

**Affiliations:** 1 Department of Pediatrics, University Hospital of Lübeck, Lübeck, Germany; 2 Department of General Pediatrics and Neonatology, Saarland University, Homburg, Germany; 3 Department of Pediatrics, University Hospital of Bielefeld, Bielefeld, Germany; 4 Department of General Pediatrics, University Children’s Hospital Muenster, Muenster, Germany; 5 Department of Neonatology, Paediatric Intensive Care, Sleep Medicine, Vest Children´S Hospital Datteln, University of Witten-Herdecke, Datteln, Germany; 6 Department of Pediatrics, University of Würzburg, Würzburg, Germany; 7 Institute of Medical Microbiology, University of Lübeck and University Hospital of Schleswig-Holstein, Campus Lübeck, Lübeck, Germany; 8 Infectious Disease Clinic, University of Lübeck and University Hospital of Schleswig-Holstein, Campus Lübeck, Lübeck, Germany; 9 German Center for Infection Research, Partner Site Hamburg-Lübeck-Borstel-Riems, Lübeck, Germany; 10 Airway Research Center North (ARCN), German Center for Lung Research (DZL), Lübeck, Germany; 11 Department of Pediatric PneumologyAllergology and Neonatology, Hannover Medical School, Hannover, Germany; 12 Department of Neonatology and Pediatric Intensive Care, Perinatal Center, West Coast Hospital Heide (WKK Heide), Heide, Germany; 13 Practice for Paediatrics and Adolescent Medicine Am Klingenberg, Lübeck, Germany; 14 Practice for Paediatrics and Adolescent Medicine in Lübeck-Kücknitz, Lübeck, Germany

**Keywords:** VLBWI, Schedule delay, Preterm, Vaccination, Immunisation schedule, Vaccine hesitancy

## Abstract

**Background:**

Although very low birthweight infants (VLBWI, birthweight < 1500 g) are at increased risk of infectious diseases, they frequently receive primary vaccinations with the hexavalent vaccine (DTaP-IPV-Hib-HepB) later than recommended. Detailed data on the adherence to the hexavalent 3 + 1 immunisation schedule (at 2, 3, 4 and 11 months of age) in German VLBWI are scarce.

**Objective:**

This study evaluated the timeliness of the primary hexavalent vaccination series among VLBWI from the German Neonatal Network (GNN) and aimed at identifying risk factors and outcomes associated with delayed series completion.

**Study design and methods:**

As part of the multicentre, population-based GNN, *N* = 3,394 VLBWI born between 2009 and 2016 underwent a follow-up examination at 6 years of age, including documentation of vaccination certificates. Uni- and multivariate analyses were performed to evaluate the timely completion of the 3 + 1 schedule for hexavalent immunisation and to examine risk factors and outcomes associated with delays.

**Results:**

Despite a high overall hexavalent vaccination coverage of more than 97% by six years of age, only 3.3% (*n* = 108) of VLBWI received the hexavalent booster at the recommended age of 11 months, and 41.6% (*n* = 1,373) had completed the primary vaccination series by 15 months of age. Progressive delays were observed across subsequent doses, with the lowest schedule-adherent coverage recorded for the booster dose. A timely first hexavalent vaccination (OR 0.61; 95% CI 0.52–0.72) was protective against delayed series completion, while bronchopulmonary dysplasia (BPD) was associated with a delay of the booster beyond 15 months of age (OR 1.31; 95% CI 1.07–1.61). Timely series completion was associated with decreased risk of incomplete vaccine protection at daycare entry (OR 0.09; 95% CI 0.07–0.14) and a lower rate of parent-reported pertussis within the first 6 years of life (OR 0.47; 95% CI 0.24–0.90).

**Conclusion:**

Our data demonstrate significant delays in the primary vaccination series which were most pronounced at the booster dose and particularly affects the most susceptible infants, i.e., VLBWI with BPD. However, vulnerable preterm infants would benefit greatly from timely vaccinations to ensure optimal protection by the time they are increasingly exposed to infectious pathogens, as for instance upon entry into childcare settings.

**Supplementary Information:**

The online version contains supplementary material available at 10.1186/s40348-026-00228-1.

## Background

Preterm infants are at increased risk of contracting infectious diseases requiring re-hospitalisation due to their immunological vulnerability, which persists beyond the neonatal period into infancy and childhood [1–3]. Immunisation serves as a cornerstone of primary preventive strategies in this high-risk group. However, immunisation strategies for preterm infants are challenged by reduced immunogenicity and the need for additional doses to ensure adequate protection [4, 5]. The Standing Committee on Vaccination (STIKO) of the Robert Koch Institute (RKI) recommends a primary 3 + 1 immunisation schedule for preterm infants, which involves the administration of the hexavalent vaccine (hexaV) at 2, 3, 4, and 11 months of age, with minimum intervals of 4 weeks between the first three doses and 6 months between the third and fourth dose. While the recommendations for full-term infants were revised to a 2 + 1 schedule in 2020 (hexaV) to improve overall adherence, the 3 + 1 schedule remained in place for preterm infants [5]. The STIKO emphasizes the importance of timely vaccination in preterm infants due to their immature immune system and strongly advises completing the series at 11 months of age to ensure early protection during the highly vulnerable phase of early infancy, with completion by 15 months of age at the latest [5–7]. However, schedule delays have been repeatedly reported for preterm infants [8–13]. From an immunological perspective, the booster dose elicits a secondary adaptive immune response by reactivating memory B and T cells, resulting in higher and more durable antibody titres, improved antibody affinity, and enhanced functional activity. It thereby reinforces long-term protection that cannot be achieved by the primary series alone [14]. This is particularly important in preterm infants, whose primary immune responses after the initial doses are substantially weaker so that the additional early dose in the 3 + 1 schedule serves to raise antibody levels to protective concentrations and to compensate for the reduced T-cell help observed in this population [4, 15]. While most preterm infants achieve short-term protective antibody concentrations after the initial three doses, a subset still requires the booster dose for achieving any protection at all and for the majority it is essential to establish robust long-term immunity [16]. Frequent concerns exist among parents and healthcare providers about potential vaccine complications in VLBWI, often driven more by a perceived, rather than actual, heightened risk for adverse effects. Although transient unspecific cardiorespiratory responses such as apnoea and bradycardia may occur in VLBWI after vaccination, these are typically of limited clinical relevance and can be effectively managed through appropriate monitoring [17, 18]. Nevertheless, concerns regarding vaccine safety and immunogenicity may influence vaccination timing [19–21]. Prematurity-related morbidities, such as bronchopulmonary dysplasia (BPD), retinopathy of prematurity (ROP) and the need for surgical interventions during primary hospitalisation have been associated with vaccination delays and hesitancy among both parents and healthcare professionals. However, arguments raised to justify postponement frequently do not represent true contraindications to vaccination [22]. Hesitancy appears to increase with vulnerability and the presence of clinical complications of prematurity [23]. Nevertheless current evidence has consistently demonstrated the safety and benefits of immunisations in preterm infants [24, 25].

In Germany, data on schedule adherence in VLBWI are scarce. We previously reported delayed initial hexavalent vaccination in German NICUs, as well as low overall influenza immunisation coverage of only 26% among VLBWI [23, 26, 27]. Evidence is lacking as to whether delayed series onset compromises timely booster completion – thereby limiting vaccine-induced immune protection and leaving infants underprotected during high risk exposure periods such as entry into early child care—and whether specific prematurity related morbidities further exacerbate these delays.

Hence, within this study of the German Neonatal Network (GNN), we aimed at evaluating the timeliness of the completion of the primary hexavalent immunisation series and to identify associated risks and outcomes.

## Study design and methods

**Fig. 1:**
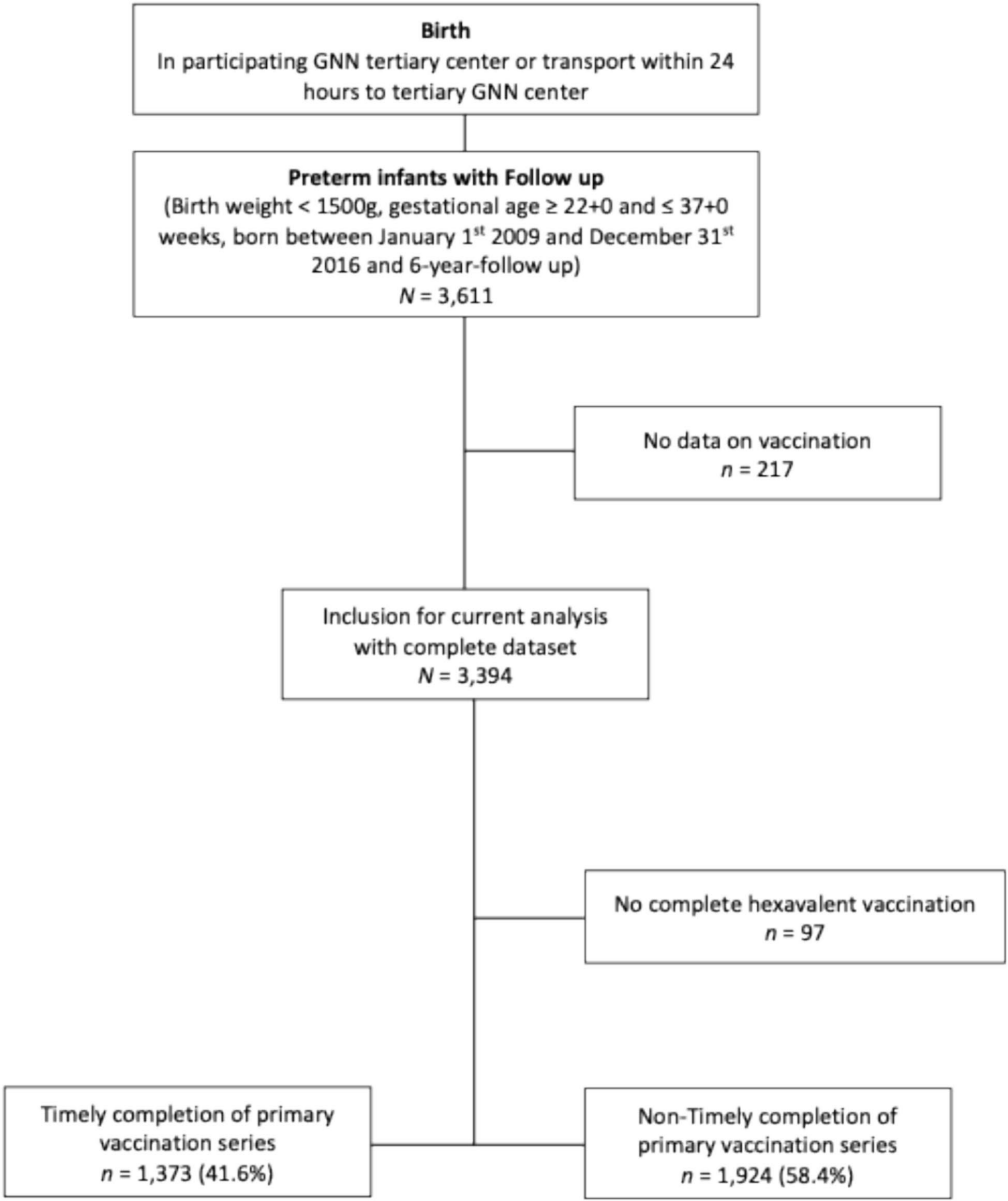
Flowchart of GNN, German neonatal network, study cohort with in- and exclusion criteria. Preterm infants with follow-up at 6 years of age (birth weight < 1500 g; gestational age gestational age ≥ 22 + 0 and ≤ 36 + 6 weeks, born between January 1 st 2009 and December 31 st 2016) were eligible for inclusion. Infants with missing vaccination data (*n* = 217) were excluded, resulting in 3,394 infants available for analysis. For the analysis of timely immunisation, only children who received all four doses of the hexavalent immunisation were included, leading to the exclusion of *n* = 97 additional cases. Timely immunisation was defined as receipt of the 4.^th^ hexavalent dose before day 450 of life (according to STIKO recommendations)

The German Neonatal Network (GNN) is a prospective, population-based, observational multicentre cohort study enrolling VLBWI at 68 neonatal intensive care units (NICU) in Germany (http://www.vlbw.de). This study included *N* = 3,394 preterm infants, born with less than 1500 g at a gestational age (GA) of 22 + 0 to ≤ 36 + 6 weeks. Infants who did not have a follow-up examination or did not provide information on vaccinations were excluded from analysis. Selected infants were born between January 1 st 2009 and December 31 st 2016. Details of the inclusion criteria are shown in Fig. 1. After obtaining informed written parental consent, each participating centre recorded a predefined dataset for each infant on the general neonatal characteristics, antenatal as well as postnatal treatment and further outcomes. Clinical data until discharge were collected by the participating NICUs. Subsequent clinical data were then monitored yearly on site by GNN staff (study nurse or paediatrician trained in neonatology). Following monitoring, patient records were transferred to the study centre (Lübeck) for processing and analysis. Annual questionnaires were sent to the parents to assess current health status and medical history from the previous year.

### Follow-up

At the age of 6 years, follow-up examinations for GNN infants were scheduled. The database was searched for potential candidates, with a focus on VLBWI born before 28 weeks of gestation. However, children born after 28 weeks, who met the general inclusion criteria were additionally recruited when examination capacity allowed and therefore were not systematically excluded. The primary care centre was contacted to schedule on-site follow-up examinations. Then, invitation letters were sent to the families that could be reached by telephone or mail and who were able to attend a follow-up appointment. A single GNN follow-up team, blinded to any interventions or complications during the primary NICU stay, carried out the examinations, including a hearing test, visual screening and spirometry. Identical instruments/equipment, which were brought to the study sites, were used for all children. Furthermore, parents were asked to complete a questionnaire covering medical history, including vaccine preventable diseases such as pertussis, current medical needs, children's social background, childcare, illnesses, general development/behaviour. All vaccinations certificates were collected and scanned. Vaccine type, number of vaccinations and dates were entered into the database. As part of this study, data were reviewed, cross-checked for accuracy, and corrected where necessary to ensure high data quality.

### Timely immunisation

Based on these STIKO recommendations, we applied two cut-off points to define timely administration of the hexavalent booster. A strict cut-off was set at the recommended time point of 11 months of age, while a broader cut-off was defined as receipt of the fourth hexavalent vaccine dose before 15 months of age (i.e., before day 450 of life). Timely administration of the first hexavalent vaccination was defined as vaccination given before day 90 of chronological age.

### Definitions

Gestational age was calculated from the best obstetric estimate from early prenatal ultrasound and obstetric examination. Small for gestational age (SGA) was defined as a birth weight less than the 10th percentile for gestational age according to sex-specific standards for birth weight by gestational age in Germany [28]. Growth velocity was defined as gain in body weight (g) per day, calculated by dividing total weight gain (weight at discharge minus weight at birth) by the number of days in hospital. BPD was diagnosed if infants still required oxygen or respiratory support at 36 weeks postmenstrual age, provided oxygen had been required for at least 28 days. Necrotising intestinal inflammation requiring surgery, was defined as a necrotising enterocolitis (NEC). Focal intestinal perforation (FIP) was defined as FIP requiring surgery classified by the attending surgeon. Any surgery was defined as all surgical interventions performed during the primary hospital stay, for example including abdominal surgery (e.g., for NEC, FIP, or stoma repositioning), correction of congenital heart defects (e.g., patent ductus arteriosus [PDA]), ROP surgery, and ventriculoperitoneal shunt placement. The Papile ultrasound criteria were used to diagnose intraventricular haemorrhage (IVH) grades I-IV [29]. Periventricular leukomalacia (PVL) was diagnosed using ultrasound imaging and was defined as white-matter brain injury including cystic degeneration of white matter near the lateral ventricles. ROP was defined as typical retinal changes diagnosed by direct fundoscopy. The composite outcome of severe complications was defined as IVH III or IV, PVL, NEC, FIP or ROP requiring intervention. In order to assess regional differences in schedule adherence, German federal states were categorised into north (Schleswig–Holstein, Hamburg, Lower Saxony, Bremen), south (Bavaria, Baden-Württemberg), east (former German Democratic Republic; GDR; Mecklenburg-Western Pomerania, Brandenburg, Berlin, Saxony, Saxony-Anhalt, Thuringia) and west (North Rhine-Westphalia, Rhineland-Palatinate, Saarland, Hesse) according to classifications previously used by *Dammann *et al. [27].

### Statistics

Data were analysed with SPSS statistics version 29.0 (IBM, NY, USA). The significance level was set at 0.05; all p-values given are two-sided. Only infants with complete data sets were included into the analysis. Pearson's chi-squared test and Mann–Whitney U test were used for univariate analyses. Logistic regression models were used to identify factors associated with delayed immunisation and incomplete immunisation at the time of childcare entry. The models were adjusted for outcome confounders and univariate group differences, including SGA, gestational age, multiple birth, ROP requiring intervention, any surgery, culture confirmed sepsis, severe IVH (grade III and IV) and BPD. A subgroup analysis was performed among infants with a primary hospital stay exceeding 60 days to examine whether the setting (inpatient vs. outpatient setting) at first-dose administration was associated with timeliness of the finalisation of the hexavalent immunisation series. Finally, an exploratory model was used to assess risk factors associated with parent-reported pertussis infection. The model included GA, BPD, presence of older siblings, childhood care and booster timeliness. Due to limited sample size no further analyses of vaccine-preventable infections such as, diphtheria or tetanus infections were performed.

### Ethics

Ethical approval for all parts of the study was obtained from the University of Lübeck Ethical Committee (vote no. 08–022, date of approval: 27th June 2008) and the committees of the other participating centres.

## Results

Follow-up examinations were performed for N = 3,611 VLBWI (for eligibility criteria see study flow chart, Fig. 1). Compared to infants without follow-up, multiple birth was overrepresented in the follow-up cohort, but no further differences in baseline clinical characteristics were observed. The final analysis included a total of *N* = 3,394 VLBWI with complete vaccination records. For analysis of timely series completion only infants with 4 doses of the hexavalent vaccination were included. Table 1 summarises baseline characteristics of the cohort. The median gestational age at birth was 28.1 weeks (IQR: 26.3–29.7) and the median birth weight was 990 g (IQR: 765–1240). Among the study population, 51.1% of the infants were male, 39.2% were multiples and 15.3% SGA. Within this cohort, 1,373 VLBWI (41.6%) had completed the full primary immunisation series by 15 months of age, whereas 1,924 (58.4%) had not received the hexavalent booster by this latest recommended timepoint for series completion according to STIKO guidelines.

**Table 1:** Clinical characteristics by completion of the hexavalent immunisation series before 15 months of age

	**Delayed completion of** **immunisation series** (*n* = 1924; 58.4%)	**Timely completion of ** **immunisation series** (*n* = 1373; 41.6%)	***p***	**Total** (*N* = 3297)
Characteristics	median (IQR)			median IQR)
Gestational age (weeks)	28.0 (26.1–29.7)	28.3 (26.6–29.9)	<.001^*#*^	28.1 (26.3–29.7)
Birth weight (g)	980 (745—1230)	990 (790–1250)	.003^*#*^	990 (765–1240)
Primary hospital stay (d)	71 (52–97)	66 (49–89)	<.001^*#*^	69 (51–93)
Age at 1 st hexavalent vaccination (d)	75 (63–101)	70 (61–89)	<.001^*#*^	73 (62–97)
Age at 2nd hexavalent vaccination (d)	119 (100–146)	110 (97–131)	<.001^*#*^	115 (99–140)
Age at 3rd hexavalent vaccination (d)	166 (140–202)	151 (133–181)	<.001^*#*^	160 (137–193)
Age at 4th hexavalent vaccination (d)	532 (483–666)	411 (386–432)	<.001^*#*^	468 (420–552)
Age at start of childhood care (d)	820 (545–1095)	790 (545–1095)	.274^*#*^	790 (545–1095)

	% (95% CI)			% (95% CI)
Gender (male)	52.2 (50.0–54.5)	49.6 (47.0–52.2)	.136	51.1 (49.4–52.8)
Multiples	39.6 (37.4–41.8)	38.7 (36.1–41.3)	.610	39.2 (37.5–40.9)
SGA	14.8 (13.2–16.4)	16.0 (14.2–18.0)	.321	15.3 (14.1–16.5)
IVH Grade 3/4	6.6 (5.6–7.8)	4.6 (3.6–5.8)	.015	5.8 (5.0–6.6)
BPD	21.9 (20.1–23.8)	16.1 (14.2–18.1)	<.001	19.5 (18.1–20.9)
Any Surgery	24.8 (22.9–26.8)	20.9 (18.8–23.1)	.009	23.2 (21.8–24.6)
ROP requiring intervention	4.5 (3.7–5.5)	3.6 (2.7–4.7)	.176	4.1 (3.5–4.8)
Timely 1 st hexavalent vaccination	66.0 (63.9–68.1)	75.9 (73.6–78.1)	<.001	70.1 (68.5–71.7)
Timely 2nd hexavalent vaccination	51.5 (49.3–53.7)	64.9 (62.3–67.4)	<.001	57.1 (55.4–58.8)
Timely 3rd hexavalent vaccination	35.8 (33.7–38.0)	49.0 (46.4–51.7)	<.001	41.3 (39.6–43.0)
Start childhood care without hexavalent booster vaccination	28.6 (26.4–30.8)	11.0 (9.3–12.9)	<.001	21.2 (19.7–22.7)
RSV immunoprophylaxis	63.7 (61.5–65.8)	59.2 (56.5–61.7)	.009	61.8 (60.1–63.4)
Influenza vaccination	24.9 (23.1–26.9)	28.6 (26.2–31.0)	.021	26.4 (25.0–28.0)
Pertussis infection	2.2 (1.5–3.0)	1.0 (0.6–1.7)	.018	1.7 (1.3–2.3)

Continuous variables are shown as median (IQR); categorial variables are shown as percentage with 95% CI: P-values for univariate analyses were derived from Chi square test and Mann–Whitney-U-Test (#). A *p*-value < 0.05 was considered statistically significant. Only infants that received all four doses of the hexavalent immunisation were included. *N* = 2,925 for analysis of pertussis infection due to lacking data. Timely vaccination of the first, second and third doses was defined as vaccination before day 90, between day 90 and 120 and between days 120 and 150 of life, respectively. Timely completion of hexavalent immunisation was defined as receipt of the 4th dose before day 450 of life

*BPD* Bronchopulmonary dysplasia, *IVH* Intraventricular haemorrhage, *ROP* Retinopathy of prematurity, *RSV* respiratory syncytial virus, *SGA* Small for gestational age (< 10th Voight percentile), Any surgery was defined as all surgical interventions performed during the primary hospital stay, *RSV* Immunoprophylaxis refers to passive immunisation against RSV with palivizumab, *IQR* Interquartile range, *CI* Confidence interval

### Delayed completion of the primary immunisation series

In this cohort of GNN preterm infants, more than 97% completed the full primary hexavalent immunisation series (*N* = 3,297) until their follow-up exam at 6 years of age. Notably, only 3.3% received the fourth hexavalent vaccination (booster) at the recommended age of 11 months. Stratified to timely finalisation of the primary immunisation series, VLBWI with a delay beyond the age of 15 months (*n* = 1,924) were characterised by only a slightly lower gestational age (28.0 weeks) and birth weight (980 g) as compared to timely immunised infants (28.3 weeks, *p* < 0.001; 990 g, *p* = 0.003). The prevalence of prematurity related morbidities was higher in the group with delayed completion, including BPD (21.9% [95% CI 20.1–23.8] vs. 16.1% [95% CI 14.2–18.1], *p* < 0.001) and any surgery during primary hospital stay (24.8% [95% CI 22.9–26.8] vs. 20.9% [95% CI 18.8–23.1], *p* = 0.009). There were no differences between the groups with regard to socio-economic and familial factors, regional differences as well as maternal origin (supplementary Table 1). Furthermore, influenza vaccination coverage was lower among infants with delayed schedule completion, whereas a higher proportion of these infants received respiratory syncytial virus (RSV) immunoprophylaxis with palivizumab. Although the delayed group reached full vaccination coverage 121 days after the timely vaccinated group, this did not translate into a correspondingly later start of daycare (*p* = 0.27). Consequently, the proportion of infants entering daycare with incomplete vaccination coverage (21.2% of the cohort) due to a missing booster dose was more than twice as high in the delayed group compared to the timely vaccinated group (28.6% vs. 11.0%, *p* < 0.001). Notably, the parent-reported incidence of pertussis within the first six years of life was higher among infants with delayed vaccination (2.2%; 95% CI 1.5–3.0) compared to those fully immunised by 15 months of age (1.0%; 95% CI 0.6–1.7; *p* = 0.018).

### Risk factors associated with delayed completion of the primary immunisation series

Timely administration of the first hexavalent vaccine was significantly associated with a reduced risk of a delayed completion of the immunisation series (OR 0.61; 95% CI 0.52–0.72), as was each additional gestational week at birth (OR 0.95; 95% CI 0.92–0.98). VLBWI with BPD had an increased risk of delayed finalisation of the primary immunisation series (OR 1.31; 95% CI 1.07–1.61). In contrast, influenza immunisation was associated with a lower risk of immunisation delays (OR 0.75; 95% CI 0.64–0.89; Table 2). In VLBWI with an age-based indication for receiving the first hexavalent dose during their initial hospital stay (primary hospital stay > 60 days, *n* = 2,121; supplementary Table 2), postponing the first vaccination to the outpatient setting was associated with an increased risk of delayed completion of the immunisation series beyond 15 months of age (OR 1.61; 95% CI 1.17–2.20).

**Table 2:** Risk factors for delayed finalisation of the primary hexavalent immunisation series

	**Adjusted OR (95% CI)**	***p*-Value**
Gestational age (per week)	0.95 (0.92–0.98)	<.001
SGA (< P10)	0.85 (0.69–1.04)	.118
Any Surgery	1.02 (0.85–1.24)	.802
BPD	1.31 (1.07–1.61)	.010
IVH Grade ≥ 3	1.26 (0.91–1.74)	.162
Influenza vaccination	0.75 (0.64–0.89)	<.001
Timely administration of 1 st dose	0.61 (0.52–0.72)	<.001

Odds ratio and 95% CIs were derived from a multivariable logistic regression model including gestational age, SGA status and relevant neonatal complications as independent variables. Timely primary immunisation was defined as receipt of the first dose of the hexavalent immunisation until the 90th day of life (according to national recommendations). Timely completion of immunisation was defined as receipt of the 4th dose before day 450 of life. A *p*-value < 0.05 was considered statistically significant

*BPD* Bronchopulmonary dysplasia, *IVH* Intraventricular haemorrhage, *SGA* Small for gestational age, *OR* Odds ratio, *CI* Confidence interval, Any surgery was defined as all surgical interventions performed during the primary hospital stay, Influenza immunisation was defined as at least one dose of the influenza vaccination

Risk factors for a delayed beginning of the 3 + 1 hexavalent immunisation series included lower gestational age, SGA, ROP requiring intervention and any surgery during the primary hospital stay (supplementary Table 3).

### Progressive delays in scheduled hexavalent immunisation

**Fig. 2:**
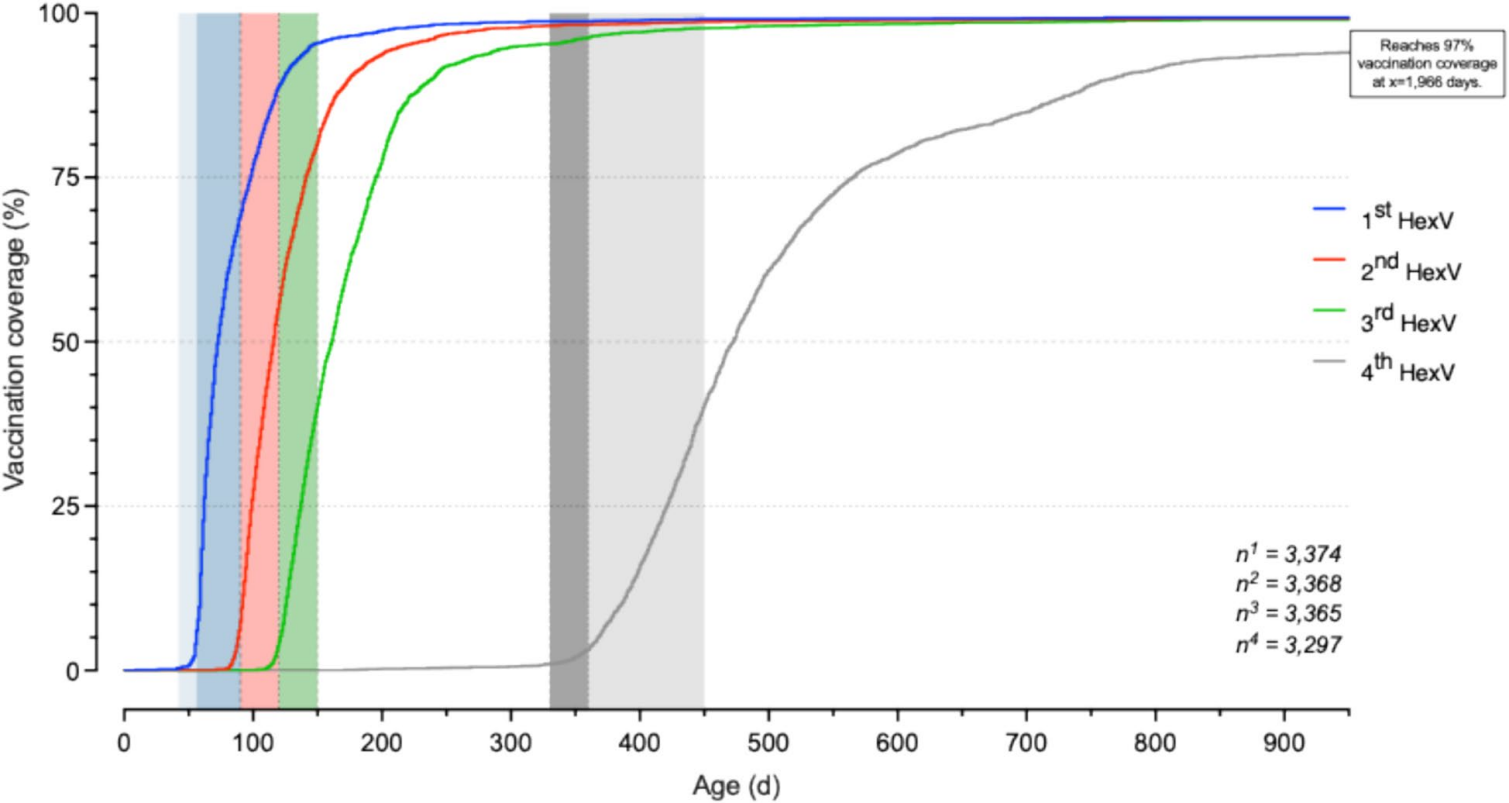
Kaplan–Meier Curves of cumulative hexavalent vaccination coverage by chronological age. Kaplan–Meier curves showing the cumulative proportion of preterm infants receiving the first (blue), second (red), third (green) and fourth (grey) dose of the hexavalent vaccine plotted against chronological age in days. *N* = 3,374 infants received the 1 st dose, *n* = 3,368 the 2nd and *n* = 3,365 the 3rd hexavalent vaccination and *n* = 3,297 received the 4th dose of the hexavalent vaccine. Shaded vertical bands indicate the recommended time windows for each dose according to the STIKO (Standing Committee on Vaccination at the RKI): 54–90 days (blue; first dose), 90–120 days (red; second dose), 120–150 days (green; third dose) and 330–360 (dark grey; fourth dose) and < 450 days of life (light grey; latest age for the completion of basic immunisation series). While coverage for the first three doses increased rapidly within the respective windows, administration of the fourth dose extended well beyond the recommended period, with 97% coverage only reached by day 1,966. To avoid distortion of the x-axis, extreme values were not mapped: *n* = 3 infants received their 1 st dose after 950 days, *n* = 2 received their 2nd dose after 950 days, *n* = 5 infants received their 3rd dose after 950 days and *n* = 106 infants received the 4th dose of the hexavalent vaccine after 950 days

Figure 2 illustrates that the first three hexavalent vaccination doses exhibited relatively steep and synchronised uptake, despite progressively increasing delays beyond their respective recommended timeframes, as indicated by the rightward shift of cumulative coverage curve intersections with the corresponding STIKO-recommended intervals. In contrast, the fourth (booster) dose displayed a markedly lower slope trajectory, with a substantial postponement extending far beyond the advised age range. The median age at administration of the first dose of the hexavalent vaccine was 73 days (IQR 62–97). Subsequent doses were administered at a median age of 115 days (IQR 99–140 days), 160 days (IQR 137–193) and 468 days (IQR 420–552) respectively. By 15 months of age more than half of the infants (58.4%) had not received a booster vaccination and 97% vaccination coverage was achieved only by day 1,966 of life.

### Incomplete vaccination coverage at start of early childhood care

A total of n = 2,851 infants attended either a daycare centre, kindergarten, nursery or childminder. The median age of starting childhood care was 790 days (IQR 545–1095 days). At the median time of entry into childhood care, 21.2% of infants had not received the booster dose of the hexavalent vaccine. VLBWI whose final vaccination was administered according to the recommended schedule were significantly less likely to enter childhood care without full vaccination coverage, regardless of the age at enrolment (OR 0.09; 95% CI 0.07–0.14).

### Immunisation protection at 6 years of age

By the age of 6 years, parent-reported history of pertussis was more frequent among infants who had not completed basic immunisation within the recommended schedule (2.2% vs 1.0%, *p* = 0.018). After adjustment for potential confounders, timely completion of the hexavalent immunisation series was associated with a reduced risk of parent-reported pertussis infection (OR 0.47; 95% CI 0.24–0.90). This analysis was exploratory given the limited number of outcome events (*n* = 51). Additionally, univariate analyses showed that VLBWI who completed their primary immunisation series according to schedule were less likely to have been hospitalised within the past 12 months at the age of follow-up (11.1% vs 14.0%, *p* < 0.022). However after adjusting for potential confounders, the association was no longer statistically significant.

## Discussion

In this large population-based GNN cohort study assessing adherence to the STIKO-recommended 3 + 1 primary immunisation schedule among German VLBWI, we observed substantial delays in both schedule initiation and series completion. Importantly, this pattern was not evenly distributed across the vaccination series: while the first three doses showed only modest delays, the delay was disproportionately concentrated on the fourth (booster) dose, representing the major driver of incomplete immunisation by 15 months of age. Only 3.3% of infants received the booster at the recommended age of 11 months, and almost 60% remained incompletely immunised by 15 months of chronological age. Risk factors for delayed completion included BPD and delayed schedule initiation. Clinically, delayed completion was associated with incomplete vaccination coverage at daycare entry and a higher parent-reported incidence of pertussis up to six years of age.

Assessing vaccination delay poses several methodological challenges. Definitions of timely versus delayed vaccination vary due to heterogeneity in national immunisation schedules, varying interpretations of age in months (ranging from 28 to 31 days) and discrepancies in manufacturer-specific vaccine recommendations that may not align with national guidelines. These inconsistencies complicate comparative analyses, hinder international comparability, and challenge the definition of clear thresholds for timely and delayed vaccination [30]. In our analysis, we adhered closely to the age-specific recommendations of the German Standing Committee on Vaccination (STIKO) for preterm infants thereby ensuring consistency with current clinical practice in Germany.

Despite differences in definitions across countries, delays within recommended immunisation schedules are consistently reported in preterm infants [8–13, 31], particularly among those with the highest medical vulnerability [23, 27]. Importantly, however, our data indicate that timeliness problems are not uniformly distributed across the series. The first three doses were only modestly delayed (median 2 weeks), whereas the booster dose showed a disproportionately large delay. This suggests that barriers differ fundamentally between initiation of immunisation — often occurring during structured NICU care — and completion of the series in outpatient settings at least nine months later.

Several structural factors likely contribute to the delay of the booster. The initial doses — if not administered during the NICU stay — generally coincide with the structured German preventive check-ups (U3, U4 and U5), facilitating timely vaccination within regularly scheduled visits [32]. In contrast, the booster requires a minimum six-month interval after the third dose and might fall between structured preventive visits, reducing automatic integration into routine care. As delays accumulate during earlier doses, alignment with the U6 examination becomes increasingly difficult, while the next scheduled preventive visit (U7) follows nearly eleven months later. Consequently, the booster is no longer reliably embedded within the structured preventive framework, rendering its administration more dependent on individual scheduling, parental initiative, and practice-level organisation. In addition, the booster is due at an age when infants experience a rising burden of respiratory infections [33, 34], leading to repeated postponement for intercurrent illnesses — particularly in outpatient care. Together, these factors create a structural vulnerability for delayed booster administration that differs from barriers affecting early vaccination in the NICU setting. This distinction may explain why delays in our cohort were largely concentrated at the end of the primary series rather than during initial doses.

From an immunological perspective, timely completion of the immunisation series is critical for several reasons. First, susceptibility to infections and infection-related complications is highest during early infancy [1, 3, 35]. Accordingly, series completion should be achieved as early as recommended (11 months of age)—yet only 3.3% of infants in our cohort received the booster by this time. Second, timely completion of the immunisation series is particularly important in preterm infants, whose vaccine responses are generally less robust than those of term infants [4, 15, 36]. While booster doses enhance antibody quality and durability in all populations, preterm infants demonstrate greater dependency on timely booster administration [4, 15, 19, 36, 37]. Notably, data from the PRIMAL consortium (Priming of Immunity at the Beginning of Life) demonstrated that without the booster dose, at least 50% of preterm infants failed to achieve long-term protective IgG levels for individual antigens, and only a small minority of 15% showed robust protection against all four vaccine components (tetanus, diphtheria, Hib, and poliovirus). Although most infants developed short-term immunity after three doses, up to 27% failed to mount protective antibody responses, depending on the antigen. In contrast, complete and timely 3 + 1 vaccination resulted in protective antibody levels in all infants [16]. These findings underscore that, in preterm infants, the booster not only provides a foundation for durable long-term immunity but also compensates for gaps in initial immune responses and thereby ensures adequate protection.

The main clinical implication of delayed booster administration is under-vaccination at daycare entry, a period of sharply increasing pathogen exposure [38], as more than half of VLBWI remained incompletely immunised at 15 months. The delays observed in our cohort resulted in over 20% of the children entering daycare incompletely immunised –– contributing to increased infection susceptibility. While causal inference cannot be established within an observational design, it remains speculative whether insufficient vaccine protection during this “window of vulnerability due to increased exposure” contributes to the twofold higher pertussis infection incidence observed among VLBWI with delayed series completion. However, this is particularly concerning considering the recent pertussis surge in Germany, with over 22,000 cases reported in 2024 – representing the highest number since mandatory reporting was introduced in 2013 [39]. These data highlight the urgent need for improving timely booster coverage in high-risk groups such as VLBWI. Pertussis remains substantially underdiagnosed, and population-level immunity is undermined by insufficient booster coverage, particularly among adolescents and adults, who serve as reservoirs of transmission to vulnerable infants [40, 41].

Another concern regarding delayed completion of the immunisation series is that it particularly affects infants with prematurity-related morbidities such as infants with BPD. However, these infants would benefit most from early protection given their increased susceptibility to severe sequelae of vaccine-preventable infections [38]. The fact that infants with BPD are usually prioritised for other vaccinations (active and passive), such as RSV [42] and influenza [27], highlights the discrepancy in risk perception, suggesting that delays in routine vaccination may not consistently be recognised as a relevant concern. The association between timely completion and uptake of optional vaccinations suggests that parental or provider awareness may play an important role in adherence behaviour.

Our observation of decreasing timeliness with subsequent vaccinations aligns with previous studies in both term and preterm populations [8, 30, 43–45]. However, prior work has primarily focused on coverage or timing of early doses. These studies have likewise demonstrated timely administration of the hexavalent booster in only about 50% of term and preterm cases, whereas overall coverage with the booster was significantly lower in preterm compared to term infants [45–47]. However, delays in vaccinations are not confined to preterm infants but represent a more pervasive problem affecting term-born infants as well. To our knowledge, this is the first study to address specific risk factors and associated consequences of delayed booster vaccinations in preterm infants in Germany, thereby identifying potential targets for improving implementation. Importantly, delaying schedule initiation emerged as a key predictor of incomplete series completion. This supports earlier observations that postponement of early doses — often related to perceived contraindications [23, 31]— may trigger cascading delays throughout the schedule [9]. Such perceptions are frequently not evidence-based, emphasizing the need for clearer guidance and consistent messaging.

The implications for implementation are relevant for both hospital and outpatient care. Early initiation during NICU stay appears critical to prevent downstream delays. Structured transition-of-care processes, improved communication at discharge, and predefined scheduling of follow-up vaccinations may help maintain continuity [48, 49]. Education of healthcare professionals and parents remains essential to reduce misconceptions regarding vaccine tolerability in preterm infants. In addition, system-level factors should be addressed. Regional differences observed in our cohort indicate that local vaccination culture and healthcare organisation may influence adherence. Targeted regional strategies and digital reminder systems may represent effective tools to improve booster uptake [50]. Ensuring consistency between hospital-based and community-based physicians is particularly important to avoid conflicting recommendations [31, 49, 51].

### Strength and limitations

The main strength of this study was the large sample size, recruitment of patients from 68 NICUs across Germany and on-site follow-up by the same neonatology-trained staff. Additionally, the availability of comprehensive clinical data from the first 6 years of life enabled a longitudinal assessment of the full primary immunisation series. However, this study accounts for several limitations such as its observational design, which only allows for post-hoc analysis and therefore merely demonstrates associations rather than proving causality. Selection bias may be present due to loss to follow-up, and differences between participating and non-participating families cannot be fully excluded. In addition, indication bias may have influenced vaccination timing, as clinically more vulnerable infants may have been intentionally delayed. Finally, specific reasons for vaccination delay were not available within the dataset.

## Conclusion

In this cohort of VLBWI, delays in primary immunisation were common and largely driven by pronounced delays in booster administration rather than early-dose initiation. As a consequence, many infants remained incompletely protected during periods of increasing pathogen exposure. These findings highlight booster timeliness as a key target for improving immunisation strategies in preterm infants. Structured transition from NICU to outpatient care, clear guidance for providers and parents, and proactive scheduling strategies may represent critical opportunities to reduce delays and improve protection in this highly vulnerable population.

## Supplementary Information

Supplementary Material 1.

## Data Availability

All data supporting the findings of this study are available within the paper and its supplementary Information.

## Abbreviations

BPD: Bronchopulmonary dysplasia
CI: Confidence Interval
DTaP: Diphtheria, Tetanus and acellular Pertussis vaccine
FIP: Focal intestinal perforation
GA: Gestational age
GDR: German Democratic Republic
GNN: German Neonatal Network
HepB: Hepatitis B
hexaV: Hexavalent vaccine
Hib: Haemophilus influenzae type b
IPV: Inactivated Poliovirus Vaccine
IQR: Interquartile range
IVH: Intraventricular Haemorrhage
NEC: Necrotising Enterocolitis
NICU: Neonatal intensive care unit
OR: Odds Ratio
PCV: Pneumococcal conjugate vaccine
PI: Preterm infant
PRIMAL: Priming of Immunity at the Beginning of Life trial
PVL: Periventricular leukomalacia
RKI: Robert Koch Institute
ROP: Retinopathy of prematurity
RSV: Respiratory syncytial virus
SGA: Small for gestational age
STIKO: Ständige Impfkommission (Standing Committee on Vaccination at the RKI)
VLBWI: Very low birthweight infant
