# Supplementary material for: Risk factors and outcomes of delayed completion of the hexavalent 3 + 1 immunisation series in German preterm infants – an observational study
Source: Mol Cell Pediatr. 2026 Apr 10;13:14. doi: 10.1186/s40348-026-00228-1 (PMC13069074; doi:10.1186/s40348-026-00228-1)
Supplement: Supplementary file 1 — Supplementary Material 1. [file 40348_2026_228_MOESM1_ESM.docx]

**Supplementary Table 1**. Socio-economic and familial factors stratified by timeliness of vaccination series completion

|  | **Non-Timely completion of**  **vaccination**  (n = 1924; 58.4%) | **Timely completion of vaccination**  (n = 1373; 41.6%) | **p** |
| --- | --- | --- | --- |
|  | **% (95% CI)** | |  |
| **No school leaving qualification (mother)** | 1.9  (1.3-2.7) | 1.8  (1.2-2.7) | .834 |
| **No school leaving qualification (father)** | 2.1  (1.4-2.9) | 1.9  (1.3-2.9) | .840 |
| **Unemployed (mother)** | 3.2  (2.5-4.2) | 2.9  (2.0-3.9) | .569 |
| **Unemployed (father)** | 2.3  (1.6-3.1) | 1.8  (1.1-2.7) | .396 |
| **Maternal descent** |  |  |  |
| **Germany** | 80.0  (78.2-81.7) | 82.4  (80.4-84.4) | .076 |
| **Other Europ. countries, incl. Russia** | 9.0  (7.8-10.4) | 7.7  (6.4-9.2) | .170 |
| **Middle East/ Turkey** | 6.0  (5.0-7.2) | 4.6  (3.6-5.8) | .072 |
| **Asia** | 1.3  (0.9-1.9) | 0.8  (0.4-1.4) | .175 |
| **Africa** | 2.4  (1.8-3.1) | 2.4  (1.7-3.3) | .981 |
| **Older siblings** | 86.8  (85.1-88.3) | 85.0  (82.9-87.0) | .002 |
|  |  |  |  |
| **Regional differences** | **% (95% CI)** | |  |
|  | **West federal states** | **East federal states** |  |
| **Timely 4^th^ hexavalent vaccination** | 41.4  (39.6-43.3) | 42.6  (38.7-46.5) | .593 |
|  | **North federal states** | **South federal states** |  |
| **Timely 4^th^ hexavalent vaccination** | 45.7  (41.7-49.8) | 35.8  (31.8-39.9) | < .001 |

**Legend**: Variables were derived from a parent questionnaire at the 6-year follow-up examination. CI, confidence interval. Categorial variables are shown as percentage with 95% CI. p-values for univariate analyses were derived from Chi square test Infants were stratified by timely completion of the primary immunisation series. A p-value < 0.05 was considered statistically significant. Timely completion of hexavalent immunisation was defined as receipt of the 4^th^ dose before day 450 of life. Eastern federal states (former German Democratic Republic; GDR) and western federal states were compared as well as northern and southern federal states to assess regional differences, federal states were categorised into north (Schleswig-Holstein, Hamburg, Lower Saxony, Bremen), south (Bavaria, Baden-Württemberg), east (former GermanDemocratic Republic; GDR; Mecklenburg-Western Pomerania, Brandenburg, Berlin, Saxony, Saxony-Anhalt, Thuringia) and west (North Rhine-Westphalia, Rhineland-Palatinate, Saarland, Hesse) based on classifications described by Dammann et al.[27]. Due to missing data n for variables varied: n = 2,870 (school leaving qualification mother), n = 2,758 (school leaving qualification father), n = 2,832 (unemployed mother), n = 2,725 (unemployed father), n = 3,350 (older siblings).

**Supplementary Table 2**. Clinical characteristics by hospital stay **≥**  60 days

|  | **Hospital stay < 60 days**  (n = 1272, 37.5%) | **Hospital stay ≥ 60 days**  (n **=** 2121, 62.5%) | **p** |
| --- | --- | --- | --- |
| **Characteristics** | **median**  **(IQR)** | |  |
| **Gestational age (weeks)** | 30.0  (29.0-31.1) | 27.0  (25.4-28.1) | < .001*#* |
| **Birth weight (g)** | 1250  (1100-1410) | 855  (680-1015) | < .001*#* |
| **Primary hospital stay (d)** | 46  (37-53) | 86  (71-109) | < .001*#* |
| **Growth velocity (g/d)** | 23.3  (20.2 – 26.7) | 21.6  (18.8-24.4) | < .001*#* |
|  | **% (95% CI)** | |  |
| **Gender (male)** | 49.4  (46.6-52.1) | 48.2  (46.1-50.3) | .503 |
| **Multiples** | 41.0  (38.3-43.7) | 37.6  (35.6-39.7) | .054 |
| **SGA** | 14.2  (12.31-16.1) | 15.9  (14.4-17.5) | .397 |
| **Severe complications** | 4.0  (3.0-5.2) | 19.2  (17.6-21.0) | < .001 |
| **ROP with Intervention** | 0.6  (0.2-1.1) | 6.2  (5.2-7.3) | < .001 |
| **NEC with surgery** | 0.8  (0.4-1.4) | 3.3  (2.6-4.1) | < .001 |
| **Any surgery** | 4.8  (3.7-6.1) | 34.4  (32.4-36.5) | < .001 |
| **BPD** | 1.6  (1.0-2.4) | 30.0  (28.1-32.0) | < .001 |
| **Timely 1^st^ hexavalent vaccination** | 58.7  (56.0-61.4) | 76.3  (74.4-78.0) | < .001 |
| **Application of first dose after primary hospital stay** | 91.1  (89.5-92.6) | 10.2  (9.0-11.6) | < .001 |
| **Timely 4^th^ hexavalent vaccination** | 44.5  (41.7-47.3) | 39.9  (37.8-42.0) | .010 |

**Legend**: BPD, bronchopulmonary dysplasia; NEC, necrotising enterocolitis; ROP, retinopathy of prematurity; SGA, small for gestational age (< 10^th^ Voigt percentile); Any surgery was defined as all surgical interventions performed during the primary hospital stay; IQR, interquartile range; CI, confidence interval. Continuous variables are shown as median (IQR); categorial variables are shown as percentage with 95% CI. p-values for univariate analyses were derived from Chi square test and Mann–Whitney-U test (#). A p-value < 0.05 was considered statistically significant. Infants were stratified by hospital stay ≥ 60 days or < 60 days and outcomes were compared accordingly. Timely vaccination of the first doses was defined as vaccination before day 90 of life. Timely completion of hexavalent immunisation was defined as receipt of the 4^th^ dose before day 450 of life

**Supplementary Table 3**. Risk factors for delayed first hexavalent vaccination in VLBWI hospitalised > 60 days.

|  | **Adjusted OR (95% CI)** | **p-Value** |
| --- | --- | --- |
| **Gestational age (per week)** | 0.80  (0.75–0.85) | < .001 |
| **SGA (< P10)** | 1.71  (1.30–2.26) | .< .001 |
| **Multiples** | 1.28  (1.02–1.60) | .034 |
| **IVH grade 3/4** | 1.29  (0.90–1.83) | .161 |
| **ROP requiring intervention** | 1.84  (1.23–2.75) | .003 |
| **Any surgery** | 1.98  (1.57–2.50) | < .001 |
| **Blood culture positive sepsis** | 1.09  (0.83–1.44) | .525 |
| **BPD** | 1.82  (1.44–2.31) | < .001 |

**Legend**: BPD, bronchopulmonary dysplasia; IVH, intraventricular haemorrhage; ROP, retinopathy of prematurity; SGA, small for gestational age; Any surgery was defined as all surgical interventions performed during the primary hospital stay; OR, odds ratio; CI, confidence interval. Odds ratio and 95% CIs were derived from a logistic regression model including gestational age, SGA status and relevant neonatal complications as independent variables. Only VLBWI, very low birth weight infants, with a primary hospital stay > 60 days were included into the analysis. Timely primary immunisation was defined as receipt of the first dose of the hexavalent immunisation until the 90^th^ day of life (according to national recommendations). A p-value < 0.05 was considered statistically significant.

**Supplementary Table 4**. Median age of vaccination

|  | **1^st^ dose**  median  (IQR) | **2^nd^ dose**  median  (IQR) | **3^rd^ dose**  median  (IQR) | **4^th^ dose**  median  (IQR) |
| --- | --- | --- | --- | --- |
| **Hexavalent** | 73  (62-97) | 116  (99-140) | 160  (137-194) | 468  (420-552) |
| **Pneumococcal** | 72  (62-98) | 116  (99-141) | 161  (137-197) | 468  (421-545) |
|  |  |  |  |  |
|  | **Timely 1^st^ dose**  %. (95% CI) | **Timely 4^th^ dose**  % (95% CI) |  |  |
| **Hexavalent** | 69.7  (68.1-71.2) | 41.6  (40.0-43.3) |  |  |
| **Pneumococcal** | 69.8  (68.2-71.3) | 41.4  (39.6-43.1) |  |  |

**Legend**: Median; IQR, interquartile range; Median age at administration of the first, second, third and fourth (booster) doses among preterm infants, presented in days of life with IQR. N = 3,374 infants received the 1^st^ dose of hexavalent vaccine, n = 3,297 received the 4^th^ dose of the hexavalent vaccine, n = 3,340 received the 1^st^ pneumococcal vaccination, n = 3,094 received the 4^th^ pneumococcal vaccination. Timely application of the first dose was defined as administration before day 90 of life and timely application of the 4th dose before day 450 of life.
